# Top-Down and Bottom-Up Proteomics Methods to Study RNA Virus Biology

**DOI:** 10.3390/v13040668

**Published:** 2021-04-13

**Authors:** Yogy Simanjuntak, Kira Schamoni-Kast, Alice Grün, Charlotte Uetrecht, Pietro Scaturro

**Affiliations:** 1 Leibniz Institute for Experimental Virology (HPI), 20251 Hamburg, Germany; yogy.simanjuntak@leibniz-hpi.de (Y.S.); kira.schamoni@leibniz-hpi.de (K.S.-K.); alice.gruen@leibniz-hpi.de (A.G.); 2 Centre for Structural Systems Biology, 22607 Hamburg, Germany; 3 European XFEL GmbH, 22869 Schenefeld, Germany

**Keywords:** affinity purification liquid chromatography coupled to mass spectrometry (AP-LC-MS/MS), flaviviruses, coronaviruses, alphaviruses, (+)RNA viruses, bottom-up proteomics, top-down proteomics, structural proteomics

## Abstract

RNA viruses cause a wide range of human diseases that are associated with high mortality and morbidity. In the past decades, the rise of genetic-based screening methods and high-throughput sequencing approaches allowed the uncovering of unique and elusive aspects of RNA virus replication and pathogenesis at an unprecedented scale. However, viruses often hijack critical host functions or trigger pathological dysfunctions, perturbing cellular proteostasis, macromolecular complex organization or stoichiometry, and post-translational modifications. Such effects require the monitoring of proteins and proteoforms both on a global scale and at the structural level. Mass spectrometry (MS) has recently emerged as an important component of the RNA virus biology toolbox, with its potential to shed light on critical aspects of virus–host perturbations and streamline the identification of antiviral targets. Moreover, multiple novel MS tools are available to study the structure of large protein complexes, providing detailed information on the exact stoichiometry of cellular and viral protein complexes and critical mechanistic insights into their functions. Here, we review top-down and bottom-up mass spectrometry-based approaches in RNA virus biology with a special focus on the most recent developments in characterizing host responses, and their translational implications to identify novel tractable antiviral targets.

## 1. Introduction

In recent years, technical advances in mass spectrometry (MS) analysis coupled with the availability of reliable MS workflows and user-friendly data analysis software triggered multiple studies investigating persistent modulation of protein abundances, post-translational modifications, and protein–protein interactions on a global cellular scale, with unprecedented sensitivity and quantitation [1,2,3,4]. Similarly, structural MS, especially native top-down MS, has shown the ability to shed light on mechanisms regulating (dis-) assembly of viral particles and their reactivity to environmental stimuli [5,6]. Indeed, the achieved precision and applicability of top-down MS to the study of dynamic processes now complements classical structural biology techniques such as electron cryo microscopy (cryo-EM) [7] or crystallography [8].

Since the introduction of mass spectrometry (MS)-based methods in proteomics a few decades ago, the technology has seen a tremendous increase in popularity. These approaches reached a level of maturity that now makes them accessible for researchers outside the mass spectrometry community, paving the way for a golden era of MS-based methods in virology. Amongst others, MS helped to elucidate elusive aspects in replication mechanisms using global or interaction proteomics, revealing the importance of post-translational modifications (PTMs) [9,10,11]. Finely tuned viral molecular strategies were also dissected using top-down and structural MS approaches in viral enzymes and protein complexes. In the near future, MS will undoubtedly provide unique contributions to the fields of antiviral drug target identification and diagnostics, while new developments, such as single-cell proteomics, are finally pushing MS to the resolution of RNA sequencing technologies.

Two workflows have been traditionally established in MS-based proteomics: “bottom-up” and “top-down” proteomics. In bottom-up-based methods, proteins are proteolytically digested into peptides prior to their injection into the mass spectrometer, allowing an analysis of complex specimens from different biological material. Conversely, in top-down methods intact proteins are analyzed, making these workflows particularly suited for the analysis of different, co-existing post-translational modifications (PTMs) of a protein or protein complex [4,12]. Here we review current approaches, in particular proteomics-based datasets generated in the past 10 years that have been used to identify, profile, or characterize novel elusive aspects of RNA virus biology, including replication mechanisms and interaction with their hosts. Exemplary studies covering multiple RNA viruses and diverse proteomic workflows are covered, and the reader is referred to more specialized reviews.

## 2. Top-Down Methods

Top-down approaches are often combined with tandem MS methods to distinguish between protein species. By connecting multiple mass analyzers in a series, ion bundles with specific *m/z* can be selected for fragmentation (e.g., collision-induced dissociation, or CID) resulting in specific fragmentation spectra. From these, the modifications can be located and determined. Another approach to separate species in samples with microheterogeneity is charge reduction mass spectrometry. Here, alkylated ammonia or alkali metal acetate salts are being utilized. This furthermore prevents the undesired denaturation of labile proteins in the gas phase, as well as the loss of non-covalent interactions in native MS approaches, which could otherwise result in the loss of information on the protein structure [13,14,15].

In recent years, MS methods have gained more attention in virology studies. In 2019, Dülfer et al. [16] gave an overview of different structural MS methods that are being used in the analysis on a variety of viruses. Common denaturing top-down MS-based methods pose a problem in many virology studies due to the disruption of non-covalent bonds by the use of acidic water/organic solvent mixtures. This unfavorable environment leads to the denaturation of proteins in the sample. This is circumvented in native MS, which uses volatile salts as buffer surrogates, especially ammonium-based salts covering a broad pH range with different carbonate or organic counter ions, to transfer natively folded proteins into the gas phase via ESI (electrospray ionization) [16,17]. The use of modifiers like salts, small molecules, co-factors, detergents, and lipids is also possible to retain protein (-complexes) in a near native state [16,18]. The conservation of non-covalent bonds comes with advantages for the analysis of higher-order structures of proteins and protein complexes and for understanding their biological function. Classic native MS can be utilized to get a general understanding about complex stoichiometry and PTMs. Nevertheless, standard native MS falls short of really understanding the connectivity of subunits and binding location [12]. Therefore, advanced native MS workflows for in-depth analysis of the primary to quaternary structure were used. Zhou et al. named them “complex-up” and “complex-down” in 2020. By activating the protein complex, monomers and other substructures are emitted deducing composition and overall architecture. By then fragmenting the protein backbone, information about protein sequence and fold are obtained and used to localize binding interfaces and to identify directly interacting subunits [12].

By implementing ion mobility (IM) separation in the MS analysis, additional information about the 3D structure of proteins and protein complexes can be obtained [12,16,17]. Weiss et al. [19], for example, used this technique to verify the mass of HRV-A2 virions and compared its size to electron microscopy (EM) results. Besides classical MS methods, charge detection mass spectrometry (CDMS) is frequently used for large assemblies exhibiting strong microheterogeneity. Here, the *m/z* and the charge are simultaneously detected for a single particle ion allowing for direct mass calculation [20]. This, of course, only scratches the surface of possibilities in native MS. Analyzing the influence of PTMs on quaternary assemblies especially suggests itself with MS and is a major advantage of this technique over other traditional structural methods [21]. The ongoing SARS-CoV-2 pandemic showed again the importance of good and reliable methods to understand the structure and complex biological pathways of viruses. In RNA viruses, the interaction of both RNA and protein is important when it comes to the viral life cycle and, hence, identifying suitable drug targets. Here, top-down MS approaches can offer valuable insights, which are highlighted in the following.

### 2.1. RNA and Protein Complexes

There are a few examples of protein complexes of RNA viruses being analyzed with native MS showing the potential of this method. The complex formation between viral RNA and proteins is widely researched in virology. Hagan et al. [22] analyzed the RNA-protein interactions of the nucleocapsid protein of HIV-1 with respect to different RNA motifs. They also determined the stoichiometry of the protein–RNA stem loop complexes in native MS [18]. Years later, Schneeberger et al. [23] researched the binding of rev arginine-rich motive (ARM) peptides to the rev response element (RRE) stem IIB RNA in HIV-1. They were able to detect 1:1 and 1:2 RNA-peptide complexes and even higher stoichiometries for the largest RNA element. The kinetics of complex formation suggested structural rearrangements when the second peptide had bound. This could be attributed to the recognition motif being dependent on stoichiometry using fragmentation data. While the first peptide initially bound to the RRE IIB stem loop, it moved aside upon association of the second peptide, resulting in both peptides now being bound to the lower stem, probably in a relay mechanism. Hence, the RNA plays a crucial role in recruiting multiple rev ARM peptides providing alternative approaches for drug design. This detailed mapping of RNA peptide interactions was only possible by native MS allowing for the mass selection of individual complexes and providing the required precision. Understanding where and how the viral RNA binds to proteins/protein complexes is a crucial part of comprehending the function of certain proteins, which can then be used to unravel the life cycle of the virus itself.

Besides RNA–protein complexes, the interaction between different proteins is also of enormous importance. Big protein “machineries” are usually heterogeneous protein complexes, with each of the protein subunits serving a different role. In a seminal study [24], the dynamic processing of the polyprotein region nsp7–10 of SARS-CoV (a virus of the *Coronaviridae* family), containing important co-factors of the main enzymes, was investigated. Native MS enabled simultaneous analysis of the processing and coordinated the release of non-structural protein (nsp) monomers by the viral protease and subsequent protein complexation. The order of processing was identified from the intermediate polyprotein products nps7–8 and nsp7–9, which provided much higher precision than e.g., SDS-PAGE (sodium dodecyl sulphate–polyacrylamide gel electrophoresis) performed for comparison. In a follow-up [25], nsp7+8 complexes from different alpha- and betacoronaviruses, including SARS-CoV-2, showed variability in stoichiometry and topology, hence pointing to distinct assembly pathways. Notably, few amino acid exchanges in nsp8 and nsp7 determine stoichiometry switching. The largest assemblies comprised hetero-trimers and -tetramers, which suggests the hexadecamer observed in SARS-CoV-2 nsp7 and nsp8 crystal structures is triggered by crystal packing. Nsp7 and nsp8 complexes have been shown to be, amongst others, vital for the RNA-polymerase (nsp12) to replicate long RNA strands. The preassembly of nsp7+8 complexes prior to docking onto nsp12 is likely necessary. The results can be used to analyze the role of the nsp7+8 complex on the polymerase complex formation in regard to anti-viral drug development. Native MS has also been used to derive the stoichiometry of polymerase assemblies, including nsp7+8 and the helicase, and select ideal conditions for subsequent cryo-EM [7,26]. To further confirm nsp7+8 stoichiometry in conventional buffers, complexes can be stabilized via a chemical labeling method called “crosslinking” followed by MS [25]. This method is another approach to gain structural information and will be further explained in the next section.

### 2.2. Chemical Labeling Mass Spectrometry Methods

In MS, there are several techniques for labeling proteins in order to obtain structural information. The idea in covalent labeling mass spectrometry (CL-MS) is that the applied reagent labels only solvent-accessible amino acids, as buried residues are protected. Then, the labeled residues can be compared in different conditions, e.g., upon binding to another protein, inducing a conformational change. There are a variety of labeling agents with different chemical properties offering different opportunities to pursue a scientific question [27,28]. One can label amino acids specifically or generally by modifying several amino acids. The specific labeling of one type of amino acid is only possible with eight of the known naturally occurring 20 amino acids. An example for specific CL-MS used for studying viruses is the study of Iacob et al. [29]. Next to site-directed mutagenesis and limited proteolysis, they used 1,2-cyclohexanedione to modify arginine residues in order to investigate hepatitis C viral glycoprotein E2 antibody binding. Mendoza et al. [28] reviewed several chemicals that are used for targeting different amino acids and explained how to conduct CL-MS properly. Ultimately, protein surface, conformations, and ligand binding sites can be investigated by chemical labeling.

**Figure 1:**
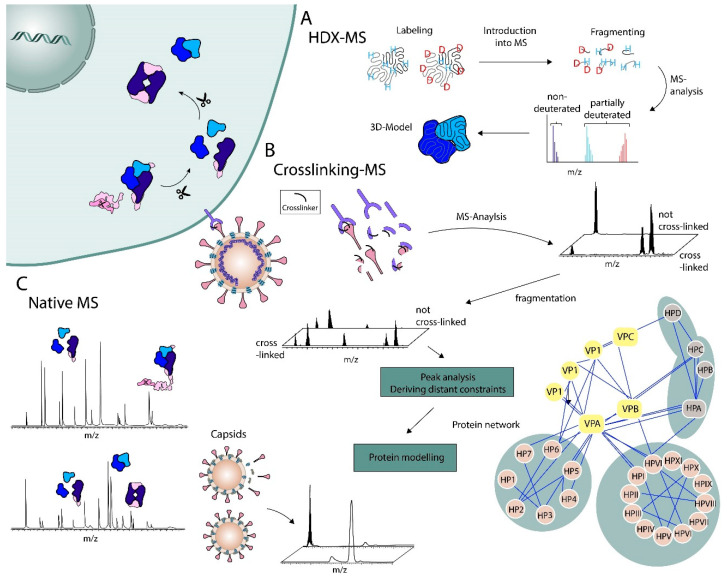
Structural mass spectrometry (MS) for RNA viruses. All depicted approaches can be conducted as top-down MS whereby exchange of hydrogen to deuterium mass spectrometry (HDX-MS) (**A**) and crosslinking mass spectrometry (XL-MS) (**B**) are currently mainly used as bottom-up techniques. For HDX-MS, the workflow starts with labeling of the natively folded protein by exchanging hydrogens to deuterium. The labeled protein is either fragmented or digested to the peptide level. Subsequent MS analyses reveal non-deuterated and partially deuterated peptides leading to constraints for a 3D model. A similar principle is used in XL-MS experiments (**B**). First, the protein complex is labeled, which can be done in both ways, in vitro and In Vivo, then the sample is fragmented or digested. Distant constraints can be deduced from every successful XL-MS experiment bringing up valuable information for computational modeling and the proposition of a structural model. In Vivo XL-MS offers the identification of a protein interaction network (bottom right) realizing the ability to unravel important virus-host association. (**C**) Native MS can determine stoichiometries of protein complexes (blue shaded) or measure whole virus capsids.

Crosslinking mass spectrometry (XL-MS) is a specific CL-MS approach to study protein complexes, in which crosslinking agents link nearby sidechains. Based on the length of the crosslinker as a constraint, 3D models can be created from distance maps [30,31]. There are different classes of crosslinkers depicting different strengths and limitations. The N-hydroxysuccinimide (NHS) esters are the most widely used mechanism creating stable bonds with primary and secondary amines such as the free N-terminus and the amino groups from lysine side chains. Mentioned limitations would be unexpected reactions with contaminant ammonium ions or unwanted reactions with serine and tyrosine residues next to the desired reactions with lysine residues [32]. Furthermore, NHS esters tend to react preferentially with tyrosine residues and free N-termini under acidic conditions [33]. This example gives an insight into aspects that need to be considered when choosing the crosslinking reagent for XL-MS experiments. Next to the reactive agent, one can design crosslinkers with different reactive groups (heterobifunctional crosslinkers) at the ends of the linking spacer arm, vary the length of the spacer arm, add a third reactive group (trifunctional crosslinker), or implement a cleavable site into the spacer arm in order to facilitate the identification of crosslinked products. This shows that there are different approaches one can choose to tackle questions of protein–protein interactions and beyond via XL-MS. Most of the common strategies and challenges in chemical crosslinking and different subsequent MS techniques are reviewed elsewhere [34,35]. XL-MS is considered a low-resolution technique and is often used as a complementary method to support other techniques, such as low resolution cryo-EM or cryo electron tomography and native MS [36]. However, this technique provides structural data sufficient to compute 3D models (cf. Figure 1). By combining derived constraints from XL-MS and integrative structural modeling, it can elucidate structural models of e.g., large protein complexes up to atomic resolution [37,38,39,40]. To conduct such experiments, it is usually necessary to purify proteins or protein complexes. Thus, structural properties are reconstituted and analyzed in vitro. For example, there are studies on coat protein topologies and epitope mapping that gave insights into the plant pathogens’ family *Luteoviridae* by using bottom-up XL-MS only [41,42].

Most of the studies quoted here used the bottom-up approach in their experiments, though such experiments can be done in a top-down approach as well. The chemical labeling with a selected cross-linker precedes MS. In order to derive constraints or in order to get a distance map it is important to obtain peptides and sequence information (cf. Figure 1). Using a top-down approach involving tandem MS, both the sequence of the chemically labeled or cross-linked peptide and the amino acid that reacted with the reactive agent are ideally detected [43]. While the bottom-up approach is more common, the top-down approach has its benefits. For instance, the protein assembly is known, which is why this is applicable to heterogeneous assemblies, and why modified amino acids can be pinpointed more easily in homodimers. Ultimately, XL-MS enables the identification of interactions between proteins, subunits, nucleic acids, and lipids.

One of the recent developments of XL-MS is In Vivo chemical crosslinking, facilitating PTM analysis or the analysis of large proteins/protein complexes that are difficult to purify. Strikingly, a more comprehensive overview of protein interaction networks can be obtained, including the identification of novel virus–host interactions [38] (cf. Figure 1). DeBlasio et al. [42] provided the first report of a host-pathogen protein interaction network of an exemplary potato leaf roll virus unraveled by XL-MS [1,42]. In Vivo XL-MS also reduces artifact-like states and provides a more accurate description of interactions in a native environment. For instance, an In Vivo crosslinking approach revealed novel host proteins bound to Dengue virus (DENV) genomic RNA that are important in RNA replication or novel RNA-binding proteins important for multiple flaviviruses such as DENV and Zika virus (ZIKV) [44,45]. In Vivo RNA–protein interaction labeling techniques are especially interesting for RNA viruses since there are essential RNA-binding proteins in the viral life cycles. An example is the identification of 309 host proteins that bind to the RNA of SARS-CoV-2 during infection. Novel proteins were found that are involved in the regulation of SARS-CoV-2 pathogenicity, and a functional connection between viral-specific proteins and mitochondria identified. Thus, In Vivo XL-MS promotes the understanding of viral mechanisms and pathogenesis, paving the way for new potential therapeutic targets [46].

In general, XL-MS techniques are widely used but little has been done yet in the context of ssRNA viruses. Due to the current pandemic, SARS-CoV-2 studies are evoking increased interest, giving rise to chemical crosslinking datasets, especially in the context of protein-RNA binding and host-pathogen association unravelling a more detailed picture in COVID-19 disease response [47,48]. Jack et al. [49] investigated an intrinsically disordered N-terminal region of SARS-CoV-2 nucleoprotein using XL-MS as complementary data to previous cryo-EM structures [1,49]. Next to cryo-EM, native MS and small angle X-ray scattering are also commonly combined with XL-MS. Chanthamontri et al. [50] combined these techniques and unraveled the shape and orientation of the Ebolavirus VP 35 oligomer that plays a critical role in RNA synthesis and host immune evasion. The latest findings using XL-MS show how valuable this technique can be for structural analyses in the context of ssRNA viruses. Especially for In Vivo chemical crosslinking, a bright future can be foreseen revealing new protein interactions important for host–pathogen association. However, one of the downsides of this technique draws attention to another one, since covalent labeling and crosslinking can both perturb protein structures due to reactions with relatively large compounds.

Chemical labeling can also distort the picture of protein structures when the experiments are not carefully conducted In Vivo. There are less perturbing strategies using the same principle, namely small labels, such as in the reversible exchange of hydrogen to deuterium (HDX) [28]. HDX coupled to MS can also provide structural information by probing the exchange from backbone amide hydrogen to deuterium, which highly depends on engagement in hydrogen bonding and secondary structure elements (Figure 1). Thus, it can especially supply local structural information, dynamics, and conformational changes [51,52,53,54]. Ultimately, this method can identify which and how amino acids are involved in ligand binding, and provides information on conformational changes upon complex formation [55]. HDX is suitable for basic structural research on viruses. For instance, on molecular mechanisms of an Ebolavirus matrix protein [56], on assembly and capsid maturation for the Brome mosaic virus and a human rhinovirus [57,58], or on the investigation of glycoproteins from diverse RNA viruses [59,60,61]. Furthermore, there was a study on hemagglutinin (HA) of influenza monitoring and characterizing structural dynamics, including relevant intermediate states during HA fusion activation on virions [62]. All mentioned studies used HDX in a bottom-up approach. It is also possible to conduct HDX as a middle- or top-down technique, but peptide generation by digestion is still more common than by fragmentation [63,64].

In conclusion, HDX and XL-MS/CL-MS are able to provide valuable structural data on proteins and protein assemblies. In the context of research on viruses, In Vivo XL-MS is especially valuable to elucidate host–pathogen associations and unknown involved proteins. Due to the fact that HDX is a gentler technique, it is particularly well-suited for the investigation of PTMs and the binding of ligands. For further reading, there are reviews that give a profound insight into the techniques and application of HDX [65] and XL-MS [35].

### 2.3. Structural Work on Viral Particles

Mass spectrometry is a powerful tool to not just analyze small molecules like metabolites or macromolecular interactions, but even to understand the high level structures of protein complexes like virus capsids. Beyond that, it has been used to investigate the architecture of various viruses [66] including (+)ssRNA viruses like tobacco mosaic virus (*Tobamovirus*) [67], foot and mouth disease virus [68], and rhinovirus (both *Picornaviridae*) [13]. To understand a virus’ life cycle, one must understand the structure of the different active parts of the virus as well as (dis)assembly mechanisms and interactions of proteins with other agents. This has been especially successful with native MS due to the nature of the method keeping the non-covalent bonds intact, as well as providing a very soft ionization method with ESI [16,69,70,71]. The architecture and dynamics of different (+)RNA viruses have been analyzed with native MS over the years. Viral particles are usually very big in size and exceed the maximal mass threshold of conventional mass spectrometers, requiring systems adapted for high molecular masses [72]. Analyzing viral particles can give insights about mechanisms and dynamics, which then can be used for drug design or screening.

An approach to this method was performed by Snijder et al. in 2013 [73] to understand the interactions between the capsid and viral genome of the picorna-like triatoma virus (TrV). In combination with atomic-force microscopy, the uncoating mechanism of the virus revealed lapsing via an intermediate state of the virion. In this intermediate state, prior to the disassembly of the capsid, the RNA is still contained. Another interesting fact discovered is that the use of viruses as nanocontainers is heavily limited by destabilizing capsid-cargo interactions and the packaging volume. These nanocontainers, when found suitable, could function as vehicles, for example, for drug delivery [73,74]. By combining different techniques, additional information can be gained on the structure of viruses [75]. Here, native MS was used in combination with bottom-up methods to analyze mass differences of sindbis virus (SINV) produced in different cells. This virus uses mosquitos as a vector. Using CDMS, a significant mass shift between a virus derived from baby hamster kidney cells (BHK) and mosquito cells (C6/36) was observed. Since virus particles from BHK cells also showed to be less infectious, glycomics and lipidomics bottom-up experiments were performed. The combination of these techniques showed that the virus derived by BHK has a different composition of lipids in the lipid bilayer. Processes like viral assembly could be affected leading to lower stability and, hence, less infectious particles. CDMS is a technique that was also used to push the boundaries in native MS looking at RNA-filled capsids of brome mosaic virus (BMV) [5]. Stability at different pHs was monitored to analyze capsid disassembly pathways. Increasing pH disrupted protein-\–protein and protein–RNA interactions of the capsid. Another approach on how to analyze highly complex virus particles was proposed by van de Waterbeemd et al. who also analyzed BMV and CCMV of the *Bromoviridae* family [76]. Obtaining high-quality spectra is challenging due to the variable content of genomic RNA, resulting in a very heterogeneous mixture of virions giving rise to complex spectra. Trimethylammonium acetate, a charge reducing additive, was used to increase the resolution by enlarging the space between charge states and distinguishing heterogeneities [5]. By rapidly increasing the pH of the sample, the disassembly of BMV was also analyzed with CDMS confirming the results from Bond et al. [5]. After a jump to higher pHs, larger capsid fragments as well as free capsid protein and complexed released RNA were detected. In addition, both groups used a similar CDMS approach and found the same mass for the virion and empty capsid, which again confirms the robustness of MS techniques. These studies show that finding the right environment at which a certain complex can be stabilized in solution, and a clever combination of available techniques, are important to get as much structural information on a complex as possible.

Additionally, a lot of structural work was performed on different noroviruses belonging to the *Caliciviridae* family. Experiments were done on stability, particle size, and the particle composition of the norovirus-like particles (NVLP) [77] of different norovirus variants [78,79], the P (protruding) domain [80,81,82], and more [82,83]. A variety of different norovirus-isolates was analyzed regarding their stability and capability to form alternative capsid sizes, showing that the stability of particles depends on pH and ionic strength. The capsid of the Norwalk virus (*T* = 3) and a closely related strain were intact at physiological pH, and higher pH triggered disassembly [77], whereas more distantly related NVLPs are resistant to alkaline treatment [79]. All variants were able to form smaller *T* = 1 particles. The truncation of the N-terminus of the major capsid protein VP1 results in exclusively smaller capsid sizes [79], showing increased alkaline stability. These smaller capsids hence show potential for vaccine design and nanotechnological approaches [6,78,79]. While other methods are not suited to analyze the dynamics and conformations of intact capsids and smaller oligomers simultaneously, native MS provides a good insight into dynamics [79,81,82].

### 2.4. Drug-Target Identification

Drug-target identification often starts in academia, where protein pathways, protein functions, and their molecular mechanisms are investigated. In Vivo XL-MS showed its potential to discover virus–host associations and pathways and, thus, can find potential interfaces for drug design [44,84] or derive a drug’s mode of action. Although bottom-up approaches were used in these studies, a top-down approach could be preferable. The latter strategy would preserve a native-like state and link protein conformation and stoichiometry to the sequence level [85].

In drug discovery, NMR and X-ray crystallography are commonly the structural methods of choice in a hit-to-lead or lead optimization strategy. The other is cell-based platforms requiring the development of primary and secondary assays and screening workflows [86]. Here, native MS and hydrogen–deuterium exchange have been used as alternative methods [55], and are regularly employed by biopharma companies. There are several reviews about the strengths of native MS in drug discovery [87,88]. Vivat Hannah et al. [88] gave an overview of native MS as a screening technique and as an approach for the characterization of protein–ligand interactions. For instance, native MS can be combined with ion mobility providing deeper insights into protein properties and assembly [87]. In contrast to protein NMR, there is no size limitation and it allows the investigation of very large complexes such as whole viruses. Thus, one could study ligands and substances that potentially bind and inhibit or disrupt viral capsid assembly.

Considering drug discovery for ssRNA viruses, this technique has not yet been the first choice. In the current pandemic, native MS was deployed as a supportive method to find potential inhibitors against the viral main protease [89,90]. In 2020, Ma et al. [89] and Sacco et al. [90] investigated potential SARS-CoV2 inhibitors of the main protease, which plays an important role in the replication of the virus. Next to enzymatic assays, they also used native MS. Another group of scientists around Alke Meents focused on screening for main protease inhibitors from approved drug repositories in order to repurpose these, which streamlines clinical trials and approvals [91]. In this study, native MS supplemented X-ray crystallography and cell-culture-based infection assays to get a complementary dataset identifying multiple compounds that deactivated the virus in cellulo. Binding sites and modes of the compounds to the protease as well as stability of the inhibitors were validated by native MS.

Moreover, Mehaffey et al. [92] used native MS and ultraviolet photo dissociation, a complementary fragmentation technique to CID, for epitope mapping of influenza viral glycoproteins. Regarding drug discovery, native MS has been used for structural studies on negative ssRNA viruses and glycan binding studies of noroviruses [81,93]. However, native MS can be more than a supportive method for studying potent inhibitors. This technique proved its ability in studies aiming for diverse targets other than viruses and viral proteins. For instance, targets such as aldose reductase, transferases, and many more [88,94,95].

Alternatively, HDX can be used in the hit-to-lead or lead optimization stage in early drug development. The investigation of the binding mode, e.g., epitope-mapping, plays a role in optimizing substances. Puchades et al. [51] showed potential drug candidates against hemagglutinin proteins of influenza and determined the epitope of four promising drugs. This technique can also be used for the identification of antibody binding and finding new target sites. Meng et al. [96] tested two single-chained antibody fragments and characterized the binding interface of one of the antibody fragments via HDX, leading to a new target for antiviral therapeutics in rhinoviruses or other enteroviruses. This method could also be used to optimize promising therapeutics or vaccines. In hepatitis C virus, immunization with potential vaccine candidates failed to give a sufficient protective response. Hereby, the key target was the conserved CD81 receptor-binding site (CD81bs) of envelope protein E2. Thorough structural investigation revealed CD81bs to be relatively flexible compared to the rest of E2, indicating a reason for the poor immune response and proposing that a structure-based design might stabilize CD81bs and thus improve the performance of the vaccine [97]. Furthermore, HDX is the method of choice when it comes to the elucidation of motile regions and domains, laying a sound foundation for drug design at flexible regions. This is how this technique facilitated a detailed structural analysis of the NS5 protein of Dengue virus serotype 3, which is an essential protein for replication [98]. Eventually, HDX is a very valuable tool to gain the structural information of flexible regions and to monitor and characterize molecular dynamics upon any binding interaction.

## 3. Bottom-Up Methods

**Figure 2:**
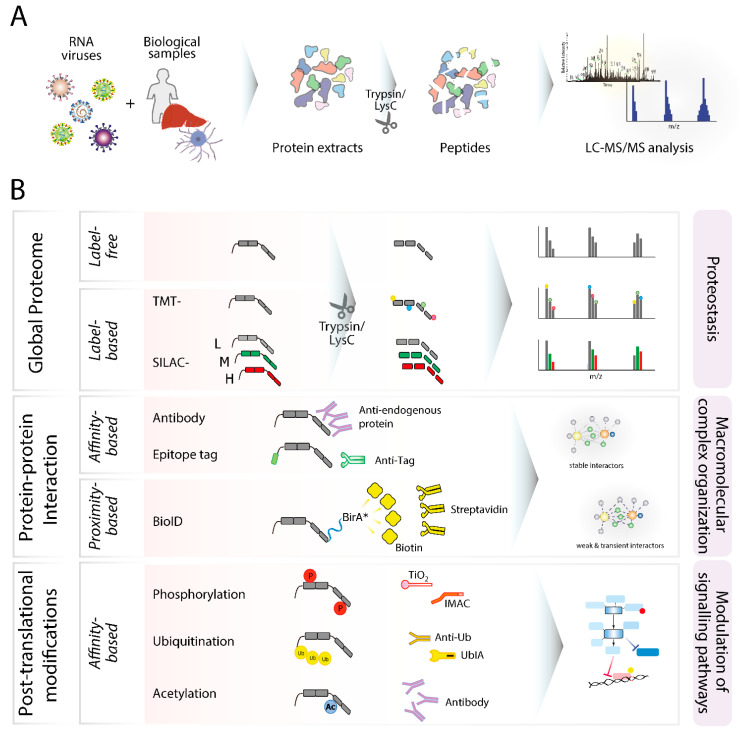
Bottom-up proteomics methods in RNA-virus biology. (**A**) Schematic work-flow of a prototypical bottom-up proteomics experiment. Virus-infected biological samples such as cells, tissue biopsies, or serum undergo protein extraction. Protein lysates are subjected to enzymatic digestion with proteases such as trypsin and/or LysC, and the resulting proteolytic peptides are analyzed by liquid chromatography coupled to mass spectrometry (LC-MS/MS). (**B**) Applications and main features of different bottom-up proteomics workflows. Schematic representation of commonly employed label-free and label-based stable isotope labeling by amino acids in cell culture (SILAC) and tandem mass tag (TMT) MS methods applied for global proteome profiling of different RNA viruses. Bottom-up proteomics workflows can be coupled with affinity purification (AP)-based methods to elucidate viral–host protein interaction networks. In addition, proximity-based methods such as BioID can be used to effectively identify transient or weak interactions between viral and host proteins. Bottom-up proteomics is also increasingly used to elucidate virus-induced modulation of signaling pathways through global analysis of post-translation modifications (PTMs), including phosphorylation, ubiquitination, and acetylation. Methods to enhance the analytical depth of PTM profiling include TiO_2_ and IMAC for phosphorylated proteins, anti-ubiquitin antibody and UbIA for ubiquitinated proteins, and anti-acetyl-lysine antibody for acetylated proteins.

In addition to top-down and structural approaches, bottom-up proteomics methods have also seen remarkable progresses in the past few years, reaching unprecedented analytical depth, sensitivity, and throughput (reviewed in [99]). For these reasons, in the last few years, bottom-up proteomics have been increasingly employed to study human pathogenic RNA viruses, shedding unprecedented light on virus-induced host perturbations at multiple levels. In bottom-up MS, a complex protein mixture obtained from biological samples of different natures (i.e., affinity-purifications, cultured cells, biopsies or autopsies, blood or serum) is proteolytically cleaved, and the resulting peptides are separated and analyzed by liquid chromatography coupled to mass spectrometry (LC-MS/MS) (Figure 2A). Depending on the research question, samples may undergo affinity purifications for the enrichment of particular proteins or modified peptides [100], or density gradients for the enrichment of specific organelles, subcellular compartments, or extracellular vesicles [101]. Bottom-up proteomics approaches can be designed to address various biological questions such as global profiling of macromolecular complexes (i.e., protein–protein interactions), proteostasis (i.e., global protein abundance), protein turnover, and PTMs (Figure 2B).

Currently available state-of-the-art LC-MS/MS set-ups allow for the reliable identification and quantification of more than 5000 proteins in very short gradients [102]. This offers a sensitive and high-throughput alternative approaching the analytical depth of RNA-based readouts such as next-generation sequencing (NGS) to investigate viral tropism and pathogenesis; the identification of therapeutic targets for antiviral development and screening for prognostic and diagnostic biomarkers; or qRT-PCR for diagnostic purposes [103].

These improvements have been the coherent result of the development of more robust and high-throughput chromatographic systems [104], the consolidation of conceptually innovative data acquisition and data-analysis approaches [105,106], as well as technical refinements or the development of completely new MS analyzers. In this respect, the introduction of innovative separation methods, semi-orthogonal to both LC and MS, such as field asymmetric ion mobility spectrometry (FAIMS) or trapped ion mobility spectrometry (TIMS), has substantially improved the detection of peptides in complex samples [107,108]. Furthermore, the latest advancements in data acquisition (i.e., data-independent acquisition, DIA; and sequential window acquisition of all theoretical fragment-ion spectra, SWATH-MS) and quantification [109,110] now facilitate the generation and optimization of spectral libraries or even no longer require them electing DIA methods to become the new gold standard in the near future.

In the following paragraphs, we provide a few recent examples of bottom-up-based methods for the study of (+)RNA virus interactions.

### 3.1. Global Proteome Profiling

Viruses often perturb global proteostasis or dynamically modulate host protein expression to degrade cellular proteins restricting virus replication, enhance the expression of host proteins critically required for productive infection, or inhibit/activate specific cellular pathways. Similarly, virus-induced host responses often entail the degradation of viral proteins or the turnover of certain cellular proteins required for effective counter defense mechanisms. Altogether, these cross talks influence viral replication fitness, viral tropism, and the cross-species transmission of zoonotic RNA viruses, and can be driven by the modulation of viral and/or cellular protein abundance.

With respect to sample preparation, label-free or label-based methods, such as stable isotope labeling by amino acids in cell culture (SILAC) and tandem mass tag (TMT), have been widely employed to study RNA viruses. Label-free proteomics is relatively inexpensive and less time-consuming compared to label-based methods, and became more popular in recent years due to the development of user-friendly software featuring reliable inter-sample normalization steps [111] and the possibility to be used on virtually any biological specimen. Conversely, label-based methods offer higher relative quantification accuracy allowing for the identification of minute differences across samples or experimental conditions (i.e., upon treatment with a small molecule or a metabolite). Furthermore, they can be used to study dynamic protein turnover or time-resolved profiling of protein abundances (i.e., pulse-SILAC-based methods), but sample preparation is laborious and only suitable for in vitro or cell-culture-based studies due to its requirement for special growth media and generation of dedicated cell lines (reviewed in [99]). For instance, SILAC requires the direct addition of stable isotope amino acids such as ^13^C or ^15^N-labeled arginine or lysine (i.e., “heavy” medium) and the natural isotopes (i.e., “light” medium) into the cell culture medium that metabolically incorporate into proteins after several cell division cycles. This approach allows for highly accurate relative quantification based on the intensity ratio of isotope-labeled peptides to unlabeled peptides [112] (Figure 2B). Additionally, label-based methods such as TMT-based approaches have been widely used to increase throughput or in clinical settings. TMT labels are usually added to the specimen at a late stage of sample preparation, making them the method of choice for clinical specimen of different biological origin and a versatile tool where high multiplexing is required [113]. However, similar to other label-based methods, this approach may suffer from so-called “ratio-compression” artefacts due to the co-fragmentation of peptides [114] and, therefore, the increased throughput is achieved at the expenses of analytical depth.

SILAC-based approaches have been widely used to profile global translation rates of host and viral proteins for multiple (+)RNA viruses such as hepatitis C virus (HCV), HIV, and DENV [115,116,117]. The most recent examples include the systematic profiling of host translation rates in SARS-CoV-2-infected cells [118]. This study identified a positive correlation between the expression kinetics of SARS-CoV-2 viral proteins and the induction of particular host translational machineries including splicing and nucleobase synthesis. Notably, translation and spliceosome inhibitors such as cyclohexamide and pladienolide-B displayed a potent inhibitory effect against SARS-CoV-2 infection in vitro [118]. Another useful application of SILAC is the possibility to couple it with orthogonal labeling methods to study the dynamics of protein expression and turnover. In a recent study, Bogdanow and colleagues elegantly combined SILAC and azidohomoalanine (AHA)-click chemistry in pulse-labelling experiments to dynamically profile the global kinetics of viral and cellular protein synthesis in influenza A virus (IAV)-infected cells [119]. While SILAC labeling allows for sensitive protein quantification, pulsed-AHA labeling allows for the selective enrichment of newly synthesized proteins using alkyne beads specifically binding AHA-conjugated proteins. This approach identified the modulation of cellular pathways involved in interferon signaling, steroid metabolism, and mitochondrial-ribosomal gene expressions as a conserved feature of both low-pathogenic avian and seasonal human H3N2 IAV strains. Interestingly, although global expression kinetics of host proteins were not differentially regulated between the two strains, this approach revealed a significantly higher expression of the M1 viral protein in seasonal human IAV-infected cells. This suggests that M1 may serve as a critical host range determinant for IAV infections across hosts [119].

TMT-based proteomics can also be used to generate quantitative and functional proteomic atlases from primary cell cultures. For instance, a recent study on HIV-infected primary human CD4^+^ T cells identified more than 600 HIV-modulated cellular proteins with functions in amino acid transports, DNA repair, and lymphocyte activation, providing a framework to study the pathophysiology of HIV-infected primary human CD4^+^ T cells at the cellular level [120]. In this instance, multiplexed TMT labeling provided a unique opportunity to monitor protein abundances in resting and activated (uninfected) T cells from the same donor, as well as control (mock-infected) T cells, allowing virus-specific effects to be distinguished from the pleiotropic changes resulting from T cell activation. This led to the identification of deregulated organic acid catabolism and lipid metabolism, indicative of an increased rate of cellular respiration, as well as the identification of novel unique HIV-Vif-regulated targets, such as FMR1 and DPH7. Moreover, TMT-based methods have recently been used to investigate the pathophysiological determinants of viral diseases. For instance, metabolomic and proteomic profiling of sera from SARS-CoV-2-infected patients identified robust upregulation of several acute phase proteins such as SAA1, SAA2, CRP, and SERPINA3 in severe COVID-19 patients requiring oxygen therapy or mechanical ventilation as compared to non-severe cases [121]. Interestingly, an independent study profiling the relative abundance of blood proteins by label-free MS/MS identified a similar proteomic signature, suggesting the comparable sensitivity of the two approaches [122]. Notably, the latter study took advantage of data-independent acquisition strategies (SWATH-DIA) and very short gradients (5 min) to screen over 100 patient samples exemplifying the great potential of label-free MS coupled with DIA, in the de novo identification of prognostic blood biomarkers to estimate severity of disease outcome.

Label-free methods can additionally be used to provide insights into the mechanisms of action of novel antiviral inhibitors. For instance, Nitazoxanide (NTZ) has been shown to exert broad antiviral activity against several RNA viruses such as HCV, Middle East Respiratory Syndrome coronavirus (MERS-CoV), and SARS-CoV-2 [123,124,125]; however, the identity of the NTZ cellular target(s) has long been elusive. Quantitative proteome analysis of NTZ-treated cells by label-free LC-MS/MS identified significant downregulation of several proteins involved in cell metabolism and RNA modifications, particularly fatty acid synthase (FASN) and heterogeneous nuclear ribonucleoprotein K (HNRNPK), suggesting that FASN and/or HNRNPK may serve as the cellular target of NTZ [126]. Interestingly, multiple RNA viruses including DENV, HCV, and respiratory syncytial virus (RSV) heavily relied on FASN activity for productive replication [127,128,129], and HNRNK was found to interact with viral RNA and promote the assembly of HCV virions [130].

### 3.2. Protein–Protein Interactions

In addition to top-down proteomics, bottom-up based approaches can also be used to systematically identify protein–protein interactions and characterize macromolecular complexes. While the first approach provides invaluable information on relative stoichiometry, 3D spatial organization, or ligand-induced rearrangements, it requires multiple optimization steps and the expression of crude or purified protein complexes prior to the analysis. Conversely, bottom-up-based methods have usually lower biochemical requirements, as protein complexes can be enriched using antibodies specific to the endogenous protein of interest, or against epitopes fused to the target of choice, in the context of intact cellular backgrounds. In this case, affinity purification (AP) usually precedes sample preparation, and can be easily multiplexed for thousands of proteins [131] or combined with chemical reactions such as biotinylation or crosslinking to capture transient or weak interactions [132,133].

Recently, global efforts to systematically profile cellular targets of viral proteins have intensified exponentially, greatly expanding the virus–host interaction landscape. Using ectopically expressed viral proteins as baits, multiple studies profiled the interactome of entire viral species including DENV, ZIKV, Ebola or Influenza virus (reviewed in [134]). For instance, by using label-free LC-MS/MS, multiple independent groups profiled the global cellular interactome of ZIKV in vertebrate and invertebrate cells, identifying cellular proteins with roles in ZIKV-induced neurodevelopmental defects [11,135,136]. Furthermore, Shah et al. identified ANKLE2, a cellular protein associated with microcephaly in humans among the NS4A-specific interactors [135,137]. By using a similar approach in the context of neural stem cells, Zeng and colleagues recently identified Dicer1 as one of the strongest ZIKV-capsid interactors, and found capsid-induced sequestration of Dicer1 sufficient to inhibit corticogenesis, likely via the downregulation of specific miRNA levels [136]. A similar LC-MS/MS approach in the context of immortalized neuroblastoma cells identified among the NS4B-specific interactor proteins with functions in neuronal degeneration, neuronal differentiation and autophagy, and axonal dysfunction such as CLN6, TMEM41b and CHP1, respectively. Notably, ectopic expression of ZIKV NS4A and NS4B in human neuronal progenitor cells has been associated with the reduced expression of cellular proteins that regulate neurogenesis and the perturbation of autophagy [11,138].

MS-driven discovery of virus-host protein–protein interactions may also streamline the identification of novel host-directed antivirals. For instance, a systematic investigation of the DENV non-structural protein 1 (NS1) cellular interactome in virus-infected cells identified RACK1 and oligosaccharyltransferase (OST) complex as conserved cellular binding partners critically required for viral RNA replication in multiple cellular backgrounds and for multiple other flaviviruses including ZIKV and West Nile virus (WNV) [139]. Pharmacological inhibitors of the OST complex display potent antiviral activity against DENV, significantly reducing NS1 secretion [139]. A similar endeavor on Uukuniemi virus (UUKV), a representative member of *Phleboviruses*, identified guanine nucleotide exchange factor 1 (GBF1) amongst the 39 cellular proteins specifically interacting with the two envelope glycoproteins G_n_ and G_c_ [140]. Functionally, mechanistic studies confirmed an essential role for the G_n_/G_c_-GBF1 interaction in supporting a post-entry step of UUKV identifying a GBF1-specific inhibitor with broad antiviral activity against UUKV and several other important RNA viruses, including chikungunya virus (CHKV), HCV, and human coronavirus 229E [140]. More recently, huge collaborative efforts attempting to uncover pan- and sub-coronavirus disease mechanisms systematically profiled the cellular interactome of multiple *Coronaviridae*, identifying several conserved interactors among SARS-CoV, SARS-CoV-2, and MERS-CoV [141]. This systematic approach allowed identifying pan-coronaviral activities, such as the PGES2-nsp7 interaction, conserved across all the three coronaviruses, as well as subnetworks unique to SARS-CoV and SARS-CoV-2. Among these, nsp6-SIGMAR1 and ORF9b-Tom70 appeared as the most promising tractable interactions with inhibitors of SIGMAR1 showing potent antiviral activity against SARS-CoV-2 in vitro [141].

In addition to traditional affinity purification-based methods providing reproducible snapshots of stable and relatively persistent macromolecular complexes, alternative approaches such as proximity-dependent biotinylation identification (BioID) have been successfully coupled to MS to identify relatively weak, transient, or indirect viral-host protein interactions (Figure 2B). BioID relies on the expression of biotin ligase(s) fused to a protein of interest allowing the biotinylation of proximal endogenous proteins. This allows for the specific enrichment of protein complexes (i.e., using streptavidin-conjugated beads), and can be coupled to relatively stringent washing conditions to remove unspecific interactors. However, its application remains challenging in cells expressing high levels of endogenous biotinylated proteins or displaying high biotin carboxylase activity [142]. A recent systematic screening of all ZIKV viral protein-associated host factors by BioID identified a higher proportion of transmembrane, nuclear, endoplasmic reticulum, and lipid droplet-associated proteins when compared to the AP-based counterpart, although the overall number of significant interactors was comparable between the two methods [143]. These results suggest that proximity-based enrichment methods might identify distinct subnetworks of interactomes, providing a complementary approach to AP-based methods in profiling extended viral-host interaction networks and secondary effector proteins. Among the most interesting developments in interaction proteomics, we note the integration of AP and BioID through the development of a single MAC-tag construct containing a twin-strep affinity tag and BirA* [132]. This approach allows for the use of the same matrix and similar procedures for both enrichment strategies, paving the way to an easier integrative characterization of a protein’s molecular context.

### 3.3. Post-Translational Modifications

In addition to global proteostasis and macromolecular complex composition, RNA viruses often deregulate post-translational modifications (PTMs) to hijack entire signaling pathways or interfere with the activity of specific effector proteins. The most commonly targeted PTMs by RNA viruses include glycosylation, ubiquitination, SUMOylation, and phosphorylation (reviewed in [144]). Irrespective of the specific PTMs of interest, bottom-up MS-based methods often entail a specific enrichment step prior to MS analysis in order to deplete the majority of the unmodified peptide counterparts and achieve higher analytical depths (reviewed in [99]).

In recent years, phosphoproteomics has seen the most dramatic technical improvements, achieving near-to-complete phosphosite coverage while requiring minute amounts of starting material. The most widely used methods for phosphopeptide enrichment involve the use of immobilized metal ion affinity chromatography (IMAC), metal oxide affinity chromatography (MOAC), titanium dioxide beads (TiO_2_), or antibody-based immunoaffinity purification (reviewed in [145]). Recently, iron-loaded nitrilotriacetic acid (Fe-NTA) was used in combination with DIA to profile the phosphoproteome of SARS-CoV-2-infected cells in a time-resolved fashion [146]. Analogously to other recent reports, the application of DIA methods to phosphoproteomics substantially increased analytical depth, allowing for the accurate identification and quantification of more than 16,000 phosphopeptides [146]. Interestingly, this study identified 33 new phosphorylation sites on 6 viral proteins (i.e., protein 3a, 9b, M, nsp6, N, and polyprotein 1ab), as well as profound hyperphosphorylation of growth factor receptors and several kinases involved in cell growth and cytoskeleton remodeling both in CaCo-2 and Vero E6 cells [146,147]. Among these, a significant activation of p38-related kinases such as casein kinase 2 (CK2), Ca^2+^/calmodulin-dependent protein kinase (CaMK2), and mitogen-activated protein kinases (MAPK) were identified in SARS-CoV-2 infected cells when compared to the uninfected counterpart. Phosphorylation of CK2 is likely to support viral egress or cell-to-cell spread through actin remodeling, while MAPK phosphorylation may play a role in the disease pathophysiology triggering cell cycle arrest [147]. Importantly, pharmacological inhibitors targeting these kinases displayed antiviral properties against infection of SARS-CoV-2 and other RNA viruses including DENV, ZIKV, and CHIKV [147,148,149]. Moreover, blocking PI3K, a downstream effector of growth factor receptor signaling, inhibits SARS-CoV-2 replication in vitro [146]. IMAC- and TiO_2_-based enrichment methods were used to systematically study the impact of IAV infections to the phosphoproteome of macrophages and lung epithelial cells [150,151,152]. Among the 1113 differentially phosphorylated proteins, IAV infection modulated proteins involved in the ubiquitin/proteasome pathway, antiviral responses, MAPK, and cyclin-dependent kinase (CDK)-signal transduction [150]. Notably, IAV entry was sufficient to trigger the phosphorylation of several kinases including CDKs and GRK, and further functional assays revealed a critical role for GRK2 activation in IAV uncoating. Notably, GRK2 specific inhibitors significantly [153,154] restrict IAV replication in vitro and IAV morbidity In Vivo [150,152]. TiO_2_-based phosphopeptide enrichment has also been used to profile infection by neurovirulent flaviviruses such as WNV and Japanese encephalitis (JEV) [153,154]. In this case, global profiling of the phosphoproteome identified induction of inflammation-associated pathways as one of the main hallmarks of viral infection. WNV infection increases the level of phosphorylated glycogen synthase kinase-3 beta (GSK3B), bifunctional polynucleotide phosphatase/kinase (PNKP), and retinoblastoma 1 (RB1). This, in turn, elevates the production of pro-inflammatory cytokines including IL-1β, IL-6, and TNF-α in WNV-infected glial U251 cells [153]. Analysis of enriched kinase substrate motifs also revealed phosphorylation of c-Jun N-terminal kinase 1 (JNK1) in JEV-infected glial U251 cells, exacerbating JEV-induced inflammation through the production of TNFα, IL6, CCL2, MMP3, and MMP9. Importantly, JNK signaling inhibitor greatly reduces the production of inflammatory cytokines in the brain and lethality in JEV-infected mice [154].

In addition to phosphorylation, the ubiquitination of specific cellular targets and viral proteins has often been associated with RNA virus infections in vitro and In Vivo [155,156]. For instance, the non-structural protein NS2 of HCV is specifically ubiquitinated by MARCH8, a RING-type E3 ligase, and this PTM was shown to be critically required for HCV envelopment, presumably supporting the binding of NS2 to HRS, an ESCRT-0 component [156]. Interestingly, MARCH8 expression was also critically required for productive DENV and ZIKV infections. Systematic profiling of ZIKV-infected trophoblasts by AP-LC-MS/MS also revealed the specific ubiquitination of viral proteins [155]. In this case, the ubiquitination of two distal lysine residues of the envelope protein was essential for viral replication in mammalian cells, and dispensable in mosquito-derived cell lines in vitro, suggesting a potential involvement in interspecies transmission. Furthermore, the ubiquitination of E protein may play a role in ZIKV tissue tropism, as it is required for viral replication particularly in the placental, testicular, and liver cells. Although profiling of ubiquitinated substrates by MS provides critical insights into potential targets of cellular ligases, the broader mechanistic consequences of ubiquitination on protein–protein interactions and altered cellular functions are often elusive. To overcome this problem, Zhang and colleagues devised an efficient strategy to enrich both ubiquitinated proteins and potential target substrates and interactors. This approach, called “ubiquitin interactor affinity enrichment–mass spectrometry” (UbIA-MS), uses chemically synthesized non-hydrolysable diubiquitin as bait for affinity purification, and has proven extremely effective in profiling ubiquitinated proteins and identify their cellular interactomes in various cell types [157].

An emerging PTM still vastly understudied in the context of RNA virus replication and virulence is protein acetylation. Until recently, no specific enrichment methods have been developed to systematically increase the relative abundance of acetylated lysine residues prior to MS, hence their identification mostly relied on the traditional AP of target proteins (or protein complexes) and targeted spectral searches. For instance, a recent study focusing on the IAV viral nucleoprotein (NP) identified multiple acetylation sites including K77, K113, and K229, playing an important role in multiple steps of the viral replication cycle [158]. In addition, acetylation of the K108 residue of NS1 protein of IAV has been shown to play an important role in the subcellular localization of NS1 and modulation of interferon-*β* signaling, suggesting a potential role in virulence and pathogenesis [159]. Similar to ubiquitination, the broader consequences of protein acetylation in the context of viral infection and their modulation of protein–protein interactions are still elusive. Recently, an acetyl-lysine immunoaffinity purification method has been successfully applied to the systematic profiling of the cellular acetylome in viral-infected cells [160]. In this case, the quantification and identification of site-specific protein acetylation was achieved via targeted MS searches using parallel reaction monitoring (PRM) requiring the selection of predefined precursor ions for fragmentation and allowing for high resolution spectral searches [160].

Additional PTMs playing critical roles in the replication cycle of multiple RNA viruses, including viral RNA replication, virus assembly, and virus entry and attachment are glycosylations. These have been extensively studied by multiple biochemical methods, including MS-based bottom-up approaches, and have been recently comprehensively reviewed elsewhere [161,162].

## 4. Conclusions

Top-down and bottom-up proteomics have entered an era of exciting discoveries. The global SARS-CoV-2 pandemic has recently shown the great potential of MS-based research in speeding up our understanding of viral replication mechanisms, identifying the perturbation of critical host factors driving viral pathogenesis, as well as streamlining the identification of host targets for drug repurposing and enabling high-resolution structural analysis by cryo-EM. Indeed, in the near future, MS will likely assist sequencing-based methods to profile emerging virus variants providing a rapid response toolkit in research and clinical settings. The recent optimization of robust high-throughput chromatographic methods and fast sample preparation protocols has already shown great promise in different fields, and will likely also enter diagnostic and prognostic pipelines in the clinical setting for infectious diseases. So far, top-down and structural MS approaches have focused on purified assemblies, showing the potential for deducing the determinants of viral protein complex formation [25]. Moreover, the field is moving towards an analysis of crude extracts in native MS, combinations of labeling and top-down approaches, as well as in situ labelling combined with MS, allowing for the profiling of complete structural networks [38]. It is conceivable that these methods will quickly enter the virology field with a transformative effect.

The improvement in sensitivity and reproducibility allowing single cell proteomics (SCP) has just emerged, and will likely also introduce new paradigms in our understanding of virus–host interactions (reviewed in [163]). Holistically combining gene-, metabolites- and protein-centric approaches will be key to exploit the full potential of MS-based methods in virology.
